# High-risk human papillomavirus prevalence and factors associated with testing positive in women living in Montserrat

**DOI:** 10.1371/journal.pgph.0006561

**Published:** 2026-06-08

**Authors:** Caroline Harris, Tiffannie Skerritt-Flemming, Dorothea Hazel-Blake, Penny Maloney, Kimona Daniel-Bourne, John Lee, Alicia Barrasa, Orwin Mosley, Anna Tirion, Riinu Pae, Sharra Greenaway-Duberry, Petra Manley

**Affiliations:** 1 UK Field Epidemiology Training Programme, UK Health Security Agency, London, United Kingdom; 2 Montserrat Ministry of Health and Social Services, Brades, Montserrat; 3 UK Overseas Territories Team, UK Health Security Agency, London, United Kingdom; 4 Behavioural Science and Insights Unit, UK Health Security Agency, London, United Kingdom; PLOS: Public Library of Science, UNITED STATES OF AMERICA

## Abstract

Montserrat is a UK Overseas Territory in the Caribbean without a universal human papillomavirus (HPV) vaccination programme for its population of 4,386. Montserrat operates limited colposcopy and cervical cancer screening services, therefore lacking evidence to determine the prevalence of high-risk HPV associated with cervical cancer. We aimed to determine the prevalence of high-risk HPV and factors associated with testing positive in women in Montserrat to inform preventive strategies. Using 2023 census data, we applied random spatial sampling to select 520 buildings and recruit 208 women based on an estimated high-risk HPV prevalence of 20%, with 5% precision at 5% significance level, anticipating a 40% response rate. Resident women aged 25–64 years were invited to participate and given the choice between self-collection or clinician-administered sampling. Samples were tested with the GeneXpert assay for 14 high-risk HPV genotypes. Sampling weights were applied to reflect the age distribution in the population. Known risk factors associated with high-risk HPV positivity were collected via questionnaire (including age group) and prevalence ratios calculated using binomial regression. Of 225 eligible women, 206 (92%) consented to participate with 187 women submitting a sample (83% of those eligible). 96% opted for self-sampling. All eligible age groups were represented in the study population. Overall high-risk HPV prevalence was 14.6% (95%CI:10.2-20.6); prevalence of HPV16 was 1.9% (95%CI:0.7-5.1) and HPV18/45 was 1.6% (95%CI:0.5-5.0) respectively. Prevalence of a positive test increased with younger age and those aged 25–34 years had three‑fold greater prevalence compared to 55–64 years (PR = 3.16, 95%CI:0.93–10.7; p = 0.064). Our findings suggest that vaccine-preventable high-risk HPV is present in the female population in Montserrat, with a trend of increased prevalence in younger age groups consistent with findings from previous Caribbean Island studies. Universal or age-targeted screening and vaccination programmes should be considered. Self-sampling HPV testing was highly acceptable to women.

## Introduction

### Montserrat

Montserrat is a self-governing UK Overseas Territory located in the Caribbean with a population of 4,386 people recorded in the 2023 census [1]. There is no comprehensive population screening programme for cervical cancer in Montserrat, however some services such as colposcopy and pap smears are available with samples sent off island for analysis, taking 6–12 weeks to be processed and returned. The cost of this offshore transport is borne by the patient (unknown due to variation by testing company and changes in price over time). Prior to this study, human papillomavirus (HPV) testing was not available on island. There is no evidence available to determine the burden of HPV infection in the local population.

### Public health importance

Human papillomavirus (HPV) is a group of viruses that commonly infect humans; the majority of HPV infections are cleared by the immune system within one or two years and do not cause harm [2]. However, some HPVs (known as high-risk HPVs) can lead to cancers; most commonly cervical, but also vaginal, vulvar, penile, anal and oropharyngeal cancer, if not detected and treated in a timely way. HPV genotypes 16 and 18 are responsible for approximately 70% of cervical cancers globally [3,4] and there are an additional 11 high-risk genotypes (31, 33, 35, 39, 45, 51, 52, 56, 58, 59 and 68) [5]. Cervical screening is key to early detection and treatment that can prevent progression to cancer [2].

WHO have called for the elimination of cervical cancer, with HPV vaccination as a core pillar of this programme alongside screening and treatment [6,7]. The Gardasil 9 vaccine, currently available in many countries globally (available in England since 2022) protects against 7 cervical cancer-causing genotypes (16, 18, 31, 33, 45, 52, 58), and additionally protects against the genital warts-associated genotypes 6 and 11 [8,9]. There is no HPV vaccination programme available in Montserrat.

### Cervical screening

Cervical screening via HPV DNA testing involves the collection of cells from either the cervix or the vagina using a cytobrush or cotton swab [10]. Whilst cervical screening has typically been carried out by healthcare staff at clinic appointments, recent studies have found that the accuracy of vaginal self-swabbing is comparable to cervical sample collection undertaken by healthcare staff [11–13]. Importantly, self-swabbing has added advantages such as increasing acceptability by providing choice for women, which may increase uptake and reach to underserved communities [14].

### HPV prevalence in the Caribbean

The prevalence of HPV in Montserrat is unknown, however similar HPV prevalence studies have been carried out in other Caribbean island nations, although these are larger populations than Montserrat. A recent study in Curaçao estimated the prevalence of HPV in women at 20%, with HPV16 being the most common high-risk genotype, followed by other high-risk genotypes 35, 18 and 52 [15]. In Martinique HPV prevalence (18 high-risk genotypes) was estimated at 19% in a study conducted between 2009 and 2014 [16]. In Saint Kitts and Nevis and Saint Vincent and the Grenadines, high-risk HPV was detected in 25% and 30% of women attending clinics on the islands respectively [17]. With an unknown cervical screening rate amongst Montserrat women due to no comprehensive screening programme, the Montserrat Ministry of Health recommended a HPV prevalence study.

### Aim

We aimed to estimate the prevalence of high-risk HPV infection in Montserrat in women of screening age (25–64 years) and extrapolate the findings to inform and make recommendations to cervical screening and vaccination programmes.

## Materials and methods

### Ethics statement

Ethical approval was granted by Montserrat’s Chief Medical Officer (ref: MHSS/CMO/4/6). All participants provided written consent to participate in the study.

### Study design

A cross-sectional study was conducted using random household sampling, with recruitment taking place between 30 October 2024 and 28 March 2025.

### Study population

Females in Montserrat aged between 25 and 64 years and who had been resident in Montserrat for over 6 months were eligible to participate in the study upon invitation.

Recruitment excluded all women who had undergone a hysterectomy; those who were pregnant or less than 3 months postpartum; and those diagnosed with cervical cancer or precancer in the last 2 years or ongoing treatment with chemo- or radiation therapy.

### Sampling and recruitment

We estimated a HPV prevalence of 20% for 14 high-risk genotypes among female residents of Montserrat, informed by previous studies conducted in Caribbean small island communities [15–17]. A power calculation carried out using Epitools [18] indicated that at a 95% confidence interval and 5% margin of error, the sample size required to confidently detect a 20% prevalence was 208 participants.

Anticipating a response rate of 30–40% (a conservative estimate, and similar response rate as was seen in HPV prevalence study in Curacao [15]), the number of women to invite for participation was 520–694. The average household size in Montserrat is 2 persons [19], and so an assumption was made that there would be only one woman per household eligible to participate in the study and so clustering was not included in the sampling design. Random household sampling was undertaken using the random selection function in Quantum GIS (QGIS) on building footprints recorded during the 2023 Montserrat census [1].A sample of 520 buildings was taken for initial visits and invitations, with an additional 174 sampled (upper sample size) in the event of the sample size threshold not being met in the first round of recruitment.

The data collectors (local healthcare professionals including clinical nurses, public health nurses and medical laboratory technologists) visited randomly assigned buildings randomly selected to invite participants. Where more than one eligible woman was present in a household, all of them would be invited. Where buildings were abandoned, not containing households or with no eligible person present, the nearest house to the left was visited instead (when looking at the building). Where there was no house to the left, the next house in an anticlockwise direction was selected. In remote areas with no nearby houses to act as replacements, the building selection was discarded. For buildings with multiple households (e.g., blocks of flats), only one household was visited based on random number generation. For absent residents, attempts were made to contact that individual (where they were known to the surveyor), or a second visit was made at a later time.

Participants received a participant information leaflet outlining the purpose of study as well as background information on HPV and cervical screening. All participants signed a consent form, and data collectors ensured informed consent prior to enrolment in study.

### Questionnaire

A short questionnaire was completed for each participant to gather demographic information and other predictive factors known from literature including smoking tobacco, use of oral contraceptives, number of children, previous cervical screening and HPV vaccination status (S1 Table). Each participant was given a unique study ID number. Questionnaires were administered by data collectors during enrolment, after consent had been given.

### Specimen collection

After confirming interest in participation, specimen collection options were provided to each participant. Self-testing was the main method promoted, with the option of clinician-administered testing if preferred; preference for either method was ascertained during recruitment. A test kit for carrying out a high vaginal swab was handed out at the point of consent, for either self-testing or to be taken to a clinic appointment. Any women who agreed to self-test but did not return a sample before the end of the study were re-contacted by the study team; any women with outstanding samples at the end of the study were assumed to have declined to participate.

### Laboratory testing

All samples were tested in the main laboratory at Glendon Hospital, Montserrat. The GeneXpert assay was used for PCR detection of 14 high-risk HPV genotypes through five separate channels (HPV16; HPV18 and 45; and other high-risk genotypes (HPV31, 33, 35, 52 and 58; HPV51 and 59; and HPV39, 56, 66 and 68)), giving 3 results for HPV16; HPV18 and 45; and other high-risk HPV [20].

Negative results were provided by phone, whilst positive results were given in person at clinic appointments with a gynaecologist. Any positive results were managed and followed up by clinicians as per established clinical pathways (S1 Fig).

A repeat high vaginal swab was requested for any invalid tests, or where specimens were grossly mucoid or blood-stained.

### Data analysis

Questionnaire and laboratory data were linked using unique study IDs. The final linked dataset was manually checked against paper laboratory records prior to analysis.

The response rate was calculated for those enrolled following the initial invitation, and also for those who enrolled and subsequently submitted a sample.

Representativeness of the study population was calculated using a chi-squared test to compare the age distribution of the study population with the that of the total cervical screening-eligible population in Montserrat.

Age-weighted prevalence for each high-risk HPV genotype group was calculated including 95% confidence intervals. Sampling weights were calculated based on the sampling frame and each set of weights was calibrated to the 2023 census enumeration by age group [21].

The number of study participants and the number of positive tests were summarised by demographic characteristics, and age-weighted univariable binomial regression was used to calculate prevalence ratios, 95% confidence intervals and Wald test p-values to determine factors associated with high-risk HPV infection. The testing option selected by each study participant was summarised. All analyses were performed using R 4.3.1.

## Results

### Response to the invitation

**Fig 1:**
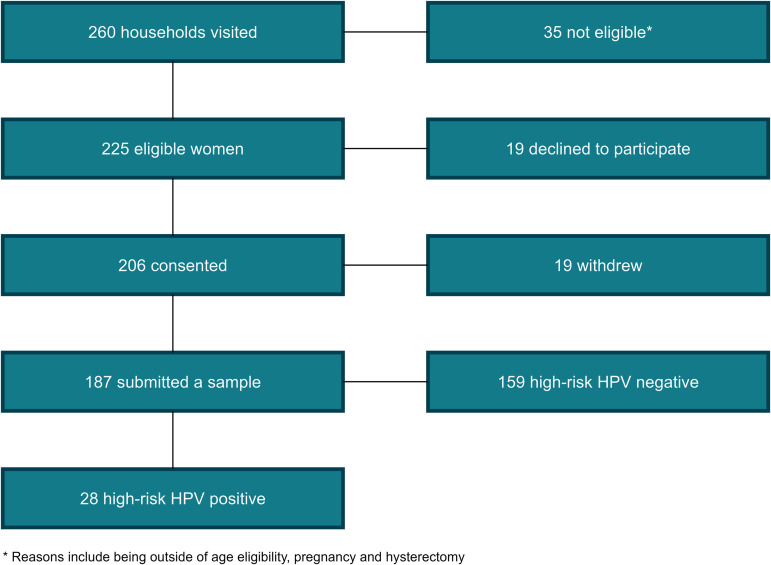
Flow chart of study recruitment and study participants‌‌.

Of 260 households approached, there were 225 eligible women and 206 (92%) consented to participate in the study. Of these women, 19 enrolled but subsequently withdrew without submitting a sample, resulting in a total of 187 women (83%) who submitted samples (Fig 1).

### Representativeness of study population

Table 1 displays the age distribution of study participants compared with the cervical screening-eligible population in Montserrat, with no significant difference between the two groups (*p* = 0.15).

**Table 1:** Comparison of Montserrat screening-age female population and study population.

Age group	Total screening-age females in Montserrat (%)^1^	Total females in study population (%)
25-34 years	22	21
35-44 years	26	33
45-54 years	26	26
55-64 years	26	20

^1^Montserrat Census 2023.

Pearson’s χ^2^= 5.3412, df = 3, p-value = 0.15.

### Prevalence of high-risk HPV

A total of 28 samples tested positive for high-risk HPV and overall age-weighted high-risk HPV prevalence was 14.6% (95% CI: 10.2 – 20.6). The highest prevalence was found for the other high-risk genotypes (11.1%, 95% CI: 7.3 – 16.6), followed by HPV16 (1.9%, 95% CI: 0.7 – 5.1) and HPV18/45 (1.6%, 95% CI: 0.5 – 5.0) (Table 2).

**Table 2:** Age-weighted high-risk HPV prevalence, overall and by genotype.

Genotype	Positive samples (n)	Prevalence (%) (95% CI)
HPV 16	4	1.9 (0.7–5.1)
HPV 18 or 45	3	1.6 (0.5–5.0)
HPV Other high-risk^1^	21	11.1 (7.3–16.6)
**Overall high-risk HPV**	**28**	**14.6 (10.2–20.6)**

^1^Other high-risk HPV genotypes includes 11 genotypes.

### Factors associated with high-risk HPV

Those aged 25–34 years had three‑fold greater prevalence of testing positive for high-risk HPV compared to those aged 55–64 years (age-weighted PR = 3.16, 95%CI: 0.93-10.7; *p* = 0.064). None of the other characteristics measured were significantly associated with high-risk HPV positivity. Notably, the three women in the study population who had received an HPV vaccine all tested negative for high-risk HPV (Table 3).

**Table 3:** Description of study population and age-weighted univariable analysis of characteristics associated with 14 high-risk HPV genotypes in women in Montserrat.

Characteristic	Negative	Positive	PR	p-value^1^
	n = 159 *n (%)*	n = 28 *n (%)*	(95% CI)	
**Age group**				
55–64 years	34 (21%)	3 (11%)	*Ref*	
45–54 years	44 (28%)	5 (18%)	1.26 (0.32-5.00)	0.7
35–44 years	52 (33%)	10 (36%)	1.99 (0.58-6.84)	0.3
25–34 years	29 (18%)	10 (36%)	3.16 (0.93-10.7)	0.064
**Tobacco smoking**				
No - never smoked tobacco	150 (94%)	27 (96%)	*Ref*	
Yes - either current or previous tobacco smoker	9 (6%)	1 (4%)	0.51 (0.07-3.64)	0.5
**Contraceptive use**				
No	55 (35%)	10 (36%)	*Ref*	
Yes - either current or previous user of oral contraceptives	104 (65%)	18 (64%)	0.87 (0.42-1.80)	0.7
**Number of children**				
None	16 (10%)	5 (18%)	*Ref*	
1	28 (18%)	8 (29%)	0.99 (0.37-2.65)	>0.9
2	49 (31%)	7 (25%)	0.49 (0.17-1.40)	0.2
3 or more	66 (42%)	8 (29%)	0.39 (0.14-1.11)	0.077
**Previous cervical cancer screening**				
No	32 (20%)	4 (14%)	*Ref*	
Yes (in the last 3 years)	88 (55%)	13 (46%)	1.12 (0.39-3.24)	0.8
Yes (more than 3 years ago)	39 (25%)	11 (39%)	1.66 (0.56-4.87)	0.3
**If you have previously had cervical screening, have you ever had an abnormal result?**				
No - all normal results	117 (92%)	21 (88%)	*Ref*	
Yes I have previously had an abnormal result	10 (8%)	3 (13%)	1.56 (0.52-4.67)	0.4
**Have you received a HPV vaccine?**				
Yes	3 (2%)	0 (0%)	–	
No	156 (98%)	28 (100%)	–	
**Study testing option**				
Attending clinic	6 (4%)	1 (4%)	*Ref*	
Self-testing	153 (96%)	27 (96%)	1.07 (0.16-7.03)	>0.9

^1^Wald test.

Almost all study participants opted for self-testing (180/187, 96%), with the proportions opting for self-testing being equal across those testing negative and those testing positive. (Table 3).

## Discussion

This study represents the first population-based estimate of high-risk HPV prevalence in Montserrat. The overall high-risk HPV prevalence in Montserratian women of 14.6% was lower but comparable to previous Caribbean Island studies [15–17].

Other Caribbean Island studies have shown varying genotype predominance [15–17] with lower prevalence of HPV16 and HPV18 compared to other global regions [4]; genotypes which are known to cause around 70% of cervical cancers globally [3,4]. In this study, the highest HPV prevalence was observed in the “other high-risk” genotypes collectively (31, 33, 35, 39, 51, 52, 56, 58, 59, 66, 68). However, the GeneXpert assay does not differentiate among these eleven genotypes. Therefore, it is unclear whether a single genotype accounted for the majority of positive results or if multiple different genotypes were detected. Comparisons with other studies’ results should be made with caution as different assays with differing genotype targets were used in each study.

Those aged 25–34 years were most likely to test positive for high-risk HPV, as found in other studies [15–17]. No other factors associated with increased prevalence were identified in this study, though it was not specifically designed to detect differences, with the sample size calculated to determine prevalence.

Only three study participants had received an HPV vaccination, therefore most of the study population would not have been protected against the genotypes contained in the vaccines [5], underlining the importance of regular screening. The introduction of a universal vaccination programme could strengthen herd immunity, whilst also providing more equitable protection against high-risk HPV for both girls and boys [5,22].

The majority of study participants opted for self-sampling, which indicates that this method of testing is acceptable to most women in Montserrat. Where possible, data collectors encouraged testing at the point of enrolment to the study and facilitated delivery of the samples to the laboratory. Some women who delayed testing at the time of enrolment subsequently dropped samples to a local clinic or directly to the laboratory, but not all. There were several advantages to offering self-sampling alongside conventional clinician-administered screening appointments, both within this study and for any future island-wide programme. The offer of self-sampling allowed women to undertake the test at their own convenience without the need for a scheduled appointment. The burden on healthcare staff was also reduced, with additional gynaecological clinics for clinician-administered sampling not being required. The option for clinician-administered sampling should still be offered for those women who would prefer this method to ensure that all women feel confident and comfortable to undergo screening.

As part of the study, HPV DNA testing was carried out on-island for the first time, with the capability to process samples on the same day or the next working day. Laboratory controls were run 2–3 times during the study period. Five members of laboratory staff were involved in testing over the study period. This rotation of staff allowed for all members to hone PCR testing and sample processing skills. On-island testing enabled the prompt return of results and arrangement of follow-up appointments for individuals who tested positive. Prior to this study, samples were sent off island for testing, with results processed and returned 6–12 weeks later. Any self-testing screening programme offered in the future should consider healthcare infrastructure, logistics and other relevant factors to increase sample return rates and reduce test kit wastage, as well as reducing the follow-up time required from healthcare staff.

There was a high enrolment rate and sample return among those invited to participate. Reasons for this may include the offer a free test, having multiple return-options to make it easier to return samples, follow-ups reminders and awareness-raising by local, respected clinicians. The study team actively followed up with study participants who did not return their samples before the end of the advertised study period. Multiple return options were offered to increase ease of sample return, but individual follow-up was still required in some instances.

Further work should be considered to investigate Montserratian women’s reasons for non-participation in screening as there was a small, but important proportion of women who either declined to enrol or withdrew from the study and did not provide a sample.

All individuals testing positive were referred to the local gynaecologist as per the protocol (S1 Fig). However, the results of colposcopy and the associated biopsies for these women were not yet available at time of writing.

The importance of establishing a cancer registry in Caribbean countries is recognised [23]. Provision of universal cervical screening in Montserrat could contribute towards a Montserrat cervical cancer registry with all data and results available to on-island health services. This would enable the monitoring of and response to cervical cancer incidence and trends in Montserrat.

### Strengths and limitations

The field work for this study encountered some challenges, particularly the inclusion of abandoned buildings or non-residential homes which required identification of replacement buildings in the field, increasing the time needed for data collection. Empty building data was not routinely collected, so it was not possible to validate the randomisation design of this study. The use of paper maps also made it difficult for data collectors to orientate themselves, so the use of online maps and GPS software is advisable for similar studies in future.

Despite the field work being carried out according to protocol, the target sample size was not reached due to the above-mentioned challenges and some attrition among participants. This allowed us to calculate prevalence but did not allow us to calculate a confidence interval with the precision we aimed for. However, the use of random sampling and the high enrolment rate amongst eligible women mean this study’s results are likely to be representative of Montserrat’s female population. It is therefore unlikely that there are any under-represented groups.

Age-weighted univariable binomial regression was used to calculate prevalence ratios, however the study was not powered to calculate adjusted prevalence ratios. Analysis was not performed by test type or region. There was only one type of test used in this study, and Montserrat has a small island population, so assessing prevalence by region would not be appropriate.

## Conclusions

The prevalence of high-risk HPV indicates a need for screening in Montserrat and the high level of acceptability for self-swabbing suggests it is possible to achieve high screening coverage without overburdening on-island clinical capacity. HPV DNA testing as the primary screening method benefits patients as it can be carried out locally, with no cost to the individual and improved timeliness of return of results compared to off-island testing. There was a high enrolment rate amongst the eligible women invited, but further work could be considered to investigate reasons for declining screening, and reasons for not returning self-testing kits to reduce wastage and staff time required for follow-up. Consideration should be given to implementing a universal HPV vaccination programme in Montserrat that would protect both men and women against the vaccine-preventable high-risk HPV genotypes.

## Supporting information

S1 Table Data collector script and study questionnaire. Script used by study data collectors and questions asked of study participants at point of recruitment to the Montserrat HPV prevalence study October 2024-March 2025. Answer options for categorical questions are also provided.
(DOCX)

S1 Fig Algorithm for primary HPV DNA screening and colposcopy triage in Montserrat. Follow up of HPV DNA testing results for participants in the Montserrat HPV prevalence study October 2024-March 2025.
(TIF)
