# Supplementary material for: High-risk human papillomavirus prevalence and factors associated with testing positive in women living in Montserrat
Source: PLOS Glob Public Health. 2026 Jun 8;6(6):e0006561. doi: 10.1371/journal.pgph.0006561 (PMC13245777; doi:10.1371/journal.pgph.0006561)
Supplement: S1 Table — Script used by study data collectors and questions asked of study participants at point of recruitment to the Montserrat HPV prevalence study October 2024-March 2025. Answer options for categorical questions are also provided. (DOCX) [file pgph.0006561.s001.docx]

**S1 Table: Data collector script and study questionnaire**

| **Script/ Questions** | **Answer options** |
| --- | --- |
| [A1] Surveyor Initials |  |
| [A2] Date of interview |  |
| [A3] Household number |  |
| [A4] Village name |  |
| **Introducing the study and checking eligibility** |  |
| [Ask to speak to all adult females in the household] |  |
| The Ministry of Health & Social Services wants to reduce cervical cancer in Montserrat, so we are doing a health study to understand how many people on the island are currently at risk. Thankfully there’s a simple test to find out whether you’re at risk, but we need your help. |  |
| Your household has been randomly selected to take part and we would like to offer cervical screening to any woman living in this household. Most women are eligible to take part; however, we just need to check a few things with you to make sure. |  |
| Check there is no reason a woman cannot take part  To be eligible they should answer YES to the following: 1. Have you lived in Montserrat for more than 6 months 2. Are you between 25 and 64 years of age  To be eligible they should answer NO to the following: 3. Have you had your womb removed 4. Are you pregnant or have given birth in the last 3 months 5. Have you had a diagnosis of cervical (pre)cancer in the last 2 years or are you being treated with chemo- or radiation therapy. |  |
| Confirm the respondent is eligible | yes no |
| **Consent** |  |
| Could we now spend some time to talk about the study? You can then decide if you want to participate – you are completely free to choose. | yes - go ahead with discussing the study  no - would not like to discuss the study  now is not the best time to discuss the study - rearrange visit |
| **Study information and consent** |  |
| Cervical cancer is a cancer that's found anywhere in the cervix, which is the opening between the vagina and the womb, sometimes called the neck of the womb.   Anyone with a cervix can get cervical cancer. It's most common in women aged between 30 and 35, but it can happen at any age. |  |
| Taking part in the study would firstly involve answering a few short questions about yourself, including your age, health and lifestyle. These questions take about 5 minutes and are confidential.   You will also need to provide a vaginal swab. This would involve giving you a test kit with some instructions on how to use it.   You can take the test yourself in your home and if convenient I could wait for the sample and deliver it to the laboratory for you or you could do it later and return it to any clinic of your choice within 24 hours of taking it.   Alternatively, a clinician could carry out the test for you, and we could arrange this for you. |  |
| The sample would be tested at the laboratory at the Glendon Hospital.   The laboratory would look for high-risk human papillomavirus, also known as HPV. This virus can cause cells to develop into cervical cancer. In the unlikely event that we found you have this virus we would refer you to a gynaecologist for further tests and early treatment. |  |
| The information we would collect (including your laboratory test) is confidential and your name would only be available to a clinician to contact you with your results.   You are completely free to participate or not as you wish.   If you do participate, you are free to stop at any time. There is no incentive to take part nor repercussion if you refuse. If you have any questions for me, I am happy to answer them. |  |
| If you’re happy to participate, I’m going to give you this leaflet with some more detailed information and instructions on how to perform the test. |  |
| If you are comfortable that you understand everything, do you consent to participate? | yes no decision to participate not made today |
| **Questionnaire** |  |
| I will now ask you for your date of birth and some health and lifestyle questions |  |
| [B3] Unique study ID |  |
| [B2] What is your date of birth? |  |
| Have you ever regularly smoked tobacco products of any kind? | no - never smoked tobacco  yes - current tobacco smoker  yes - previously smoked tobacco |
| Have you ever taken oral contraceptives? | no  yes - previous user  yes - current user, for less than 5 years  yes - current user, for over 5 years |
| How many children have you given birth to? | 1 2 3 4 5 or more None |
| Have you previously had a screening test for cervical cancer? | no, never had a cervical screening test  yes (in the last 3 years)  yes (in the last year)  yes (more than 3 years ago)  unsure |
| If you have previously had cervical screening, have you ever had an abnormal result? | no - all normal results  yes I have previously had an abnormal results  unsure |
| Have you received a HPV vaccine? | yes no |
| **Test kit** |  |
| Thank you for answering the questions. I can now give you the test.  However, there are a few reasons why you may need to delay taking the test 1. If you are on your period (you must wait 24 hours after your period has stopped) 2. If you have douched in the last 24 hours 3. If you have had sex in the last 24 hours |  |
| Self swabbing: If it is convenient then I can wait whilst you do the test now and I can deliver your sample to the laboratory. Or if you would like to do the test in your own time we would ask you to return the sample to any clinic of your choice. Samples would need to be returned within 24 hours of taking the test to get an accurate result, so please don’t do the test over the weekend. |  |
| Clinician testing: Arrange an appointment for the participant |  |
| Testing option | self-testing attending clinic |
| **Clinician testing** |  |
| Give some information on attending a clinic for an appointment  Remind them to take test kit to appointment |  |
| Thank the respondent(s) for their time. |  |
| Comment |  |

**S1 Table legend:** Script used by study data collectors and questions asked of study participants at point of recruitment to the study. Answer options for categorical questions are also provided.
