# Supplementary figures and images for: High-risk human papillomavirus prevalence and factors associated with testing positive in women living in Montserrat
Source: PLOS Glob Public Health. 2026 Jun 8;6(6):e0006561. doi: 10.1371/journal.pgph.0006561 (PMC13245777; doi:10.1371/journal.pgph.0006561)

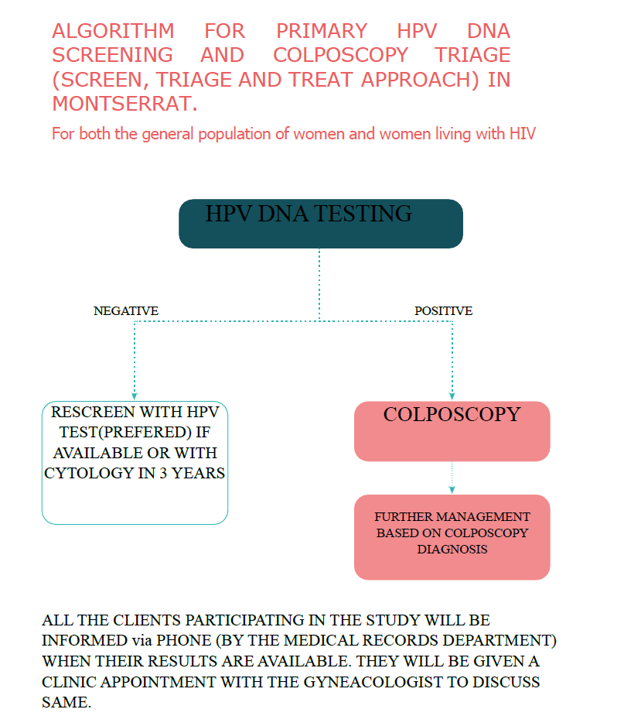

Supplement: S1 Fig — Follow up of HPV DNA testing results for participants in the Montserrat HPV prevalence study October 2024-March 2025. (TIF) [file pgph.0006561.s002.tif]
